# Inferences of drug responses in cancer cells from cancer genomic features and compound chemical and therapeutic properties

**DOI:** 10.1038/srep32679

**Published:** 2016-09-20

**Authors:** Yongcui Wang, Jianwen Fang, Shilong Chen

**Affiliations:** 1 Key Laboratory of Adaptation and Evolution of Plateau Biota, Northwest Institute of Plateau Biology, Chinese Academy of Sciences, Xining, 810001 China; 2 Biometric Research Branch, Division of Cancer Treatment and Diagnosis, National Cancer Institute, Rockville, MD 20850, USA

## Abstract

Accurately predicting the response of a cancer patient to a therapeutic agent is a core goal of precision medicine. Existing approaches were mainly relied primarily on genomic alterations in cancer cells that have been treated with different drugs. Here we focus on predicting drug response based on integration of the heterogeneously pharmacogenomics data from both cell and drug sides. Through a systematical approach, named as PDRCC (Predict Drug Response in **C**ancer **C**ells), the cancer genomic alterations and compound chemical and therapeutic properties were incorporated to determine the chemotherapeutic response in cancer patients. Using the Cancer Cell Line Encyclopedia (CCLE) study as the benchmark dataset, all pharmacogenomics data exhibited their roles in inferring the relationships between cancer cells and drugs. When integrating both genomic resources and compound information, the prediction coverage was significantly increased. The validity of PDRCC was also supported by its effective in uncovering the unknown cell-drug associations with database and literature evidences. It set the stage for clinical testing of novel therapeutic strategies, such as the sensitive association between cancer cell ‘A549_LUNG’ and compound ‘Topotecan’. In conclusion, PDRCC offers the possibility for faster, safer, and cheaper the development of novel anti-cancer therapeutics in the early-stage clinical trails.

The recent successes in precision medicine enabled us to effectively casting large-scale genomic data of cancer cells into actionable, customized prognosis and treatment regimens for individual patients. However, the systematic translation of cancer genomic data into the knowledge of tumor biology and therapeutic possibilities remains challenging^1^. Accurately predicting the cancer cell response to medication is particularly important to address this challenge and leads us to achieve the ultimate goal of personalized diagnosis and treatment. Lots of efforts have been exerted to characterize the relationships between genomic profiles and drug response^1^,^2^,^3^,^4^, and several drug response prediction algorithms have been proposed^1^,^2^,^5^,^6^. All these works highlight the substantial complexity and heterogeneity relationships between genomic alterations and drug responses. Thus, systematical approaches to integrate heterogeneous pharmacogenomics data sources are urgently needed.

In previous works, the authors attempted to predict drug responses in cancer cells based primarily on genomic features of cells that have been treated with given drugs. For example, Geeleher *et al.*, demonstrated a method for the prediction of chemotherapeutic response in patients using before-treatment baseline tumor gene expression data^7^; Venkatesan *et al.* developed a novel machine learning method to predict drug response by integrating genome-scale mRNA expression, copy number alteration and mutation profiles for nearly 1000 cancer cell line models spanning many tumor types^8^; Costello *et al.* applied the multiple kernel learning algorithm to improve drug sensitivity prediction from genomic, proteomic, and epigenomic profiling data in breast cancer cell lines^9^. Although achieving promising rersults for certain drugs, these approaches did not incorporate the information of compound and ignored the fact that structural or functional related drugs may have similar therapeutic efffect. Thus researches began to put their focuses on the development of the systematical algorithms, which predicted the responses of anti-cancer therapies in cancer cells from both genomic features and compound properties. For example, Menden *et al.* developed machine learning models to predict the response of cancer cell lines to drug treatment based on both the genomic features of the cell lines and the chemical properties of the drugs^6^; Zhang *et al.* proposed a dual-layer integrated cell line-drug network model to predict anti-cancer drug responses through incorporating similarities between cancer cells and drugs^10^.

High-throughput drug screening technologies enabled us to test of hundreds of thousands of anti-cancer therapies against a panel of cancer cell lines. The curated databases deposit the responses of thousands of cancer cells to hundreds of anti-cancer drugs, such as NCI-60^11^, the Cancer Cell Line Encyclopedia (CCLE)^1^ and Connectivity Map (CMap)^3^. These valuable information sources provide a great opportunity to understand the mechanism of cancer treatments in a comprehensive genetic background. That is, cell-drug relationships could be constructed based on high-quality measurements of drug response data. Most importantly, the understandable rules for cell-drug associations can be learned by a statistical predictor based on these associations.

Here, we developed an integrative framework to Predict Drug Responses in Cancer **C**ells (PDRCC) by dissecting the cell-drug associations in a large-scale manner. We observed that the current available data sources, including KEGG BRITE^12^, SuperTarget^13^, and DrugBank^14^, describe drug’s biological function in living cell from different levels and different aspects. For example, drug’s chemical structure provides information by the ‘structure determines function’ paradigm; ATC-code annotation provides the therapeutic effect at molecular level; Protein target hints the therapy effect at molecular level. While, multiple genomic data sources describe the alterations of cell function after treatment in diverse ways. For example, oncogene mutation and DNA copy number provide the molecular alterations at genomic level; gene expression reflects the direct changes in cells at transcriptomic level. One straightforward assumption is that drugs similar in one or more data source metrics have similar therapeutic effects on cancer cells, and cancer cells with similar genomic properties have similar responses to anti-cancer therapies. We demonstrated that drugs with similar compound chemical properties, ATC-codes, or target proteins indeed associate with response measurements in cells, and cancer cells with similar genomic properties indeed correlate with their response profiles. Then we proposed the idea to integrate heterogeneous pharmacogenomics data from both cell and drug sides. Specifically, cells and drugs were first characterized by their similarity-based profiles, and a kernel function was then defined to correlate them. Finally, the cell-drug associations were inferred by training a machine learning model, i.e., support vector machines (SVM), which is motivated by statistical learning theory^15^,^16^ and has been proven successful on many different classification problems in bioinformatics^17^. PDRCC overcomes the main difficulty to integrate heterogeneous pharmacogenomics data sources from both genomic and chemical level. Moreover, through learning the relationships between cells and drugs, PDRCC could not only predict the response of a new cell line to existing drugs, but also predict the response of an existing cell line to new drugs, thus would potentially save the cost in a drug-cell line screening. By validating our PDRCC on the well-established CCLE data, we found that all genomic and compound properties were predictive in different ways. Moreover, more cell-drug associations could be uncovered by combination of genomic and chemical properties. In addition, database and literature searching indicate that our new predictions are worthy of future experimental validation.

## Results

**Figure 1:**
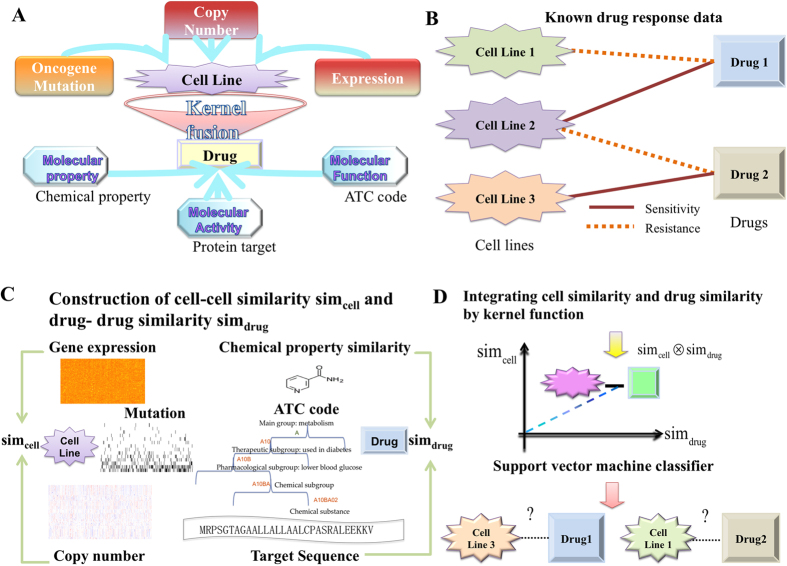
The flowchart of PDRCC. (**A**) The schematic plot for our PDRCC method. PDRCC applied the kernel method to integrate multiple information about cell, including oncogene mutation, DNA copy number, and mRNA expression, and multiple information about drug, including compound molecular properties, ATC-code, and drug side-effect, to detect the interactions between cells and drugs. (**B**) Collecting known relationships between cells and drugs as gold standard positives in a bipartite graph. (**C**) Calculating cell-cell and drug-drug similarity by genomic data of cells and chemical and therapeutic properties of drugs. (**D**) Relating the similarity among cells and similarity among drugs by Kronecker product kernel, and applying SVM-based algorithm to predict the unknown associations between cells and drugs.

Based on the assumption that cancer cells with similar genomic profiles are supposed to have similar responses to anti-cancer drugs, and anti-cancer drugs with similar chemical or therapeutic properties are hypothesized to have similar inhibition effects on cancer cells, we developed a systematically integrative method, called as PDRCC, to infer drug response in human cancer cell lines based on kernel fusion of heterogeneous pharmacogenomics data (Fig. 1A). Specifically, we first constructed bipartite graph by known drug responses in cancer cells. The two kinds of nodes in bipartite graph represent drugs and cell lines, respectively. The edges between cells and drugs represent the relationships among them, defined as either sensitivity or resistance (Fig. 1B). Then we applied compound molecular descriptors, target proteins, and ATC-codes to measure the similarity among drugs, introduced oncogene mutation, DNA copy number, and mRNA expression to quantify the similarities among cancer cells, and defined a Kronecker product kernel to correlate with them (Fig. 1C). Finally, a support vector machine was utilized to predict the unknown relationships between cells and drugs (Fig. 1D). The PDRCC was validated on the well-established CCLE data, which contains 8-point dose-response curves for 24 compounds across 504 human cancer cell lines.

### Correlation analysis shows all cancer genomic data and compound information sources are predictive

Here, to predict the associations between cancer cells and anti-cancer therapies, we integrated heterogeneous pharmacogenomics data from both cell and drug sides. Therefore, first of all, we would like to check whether each single data source is predictive or not. To this end, we correlated cancer genomic data with their response profiles, and correlated drug chemical and therapeutic properties with their inhibition effects. We hope that cells with similar genomic features have similar responses to drugs, and drugs with similar chemical or therapeutic properties exhibit similar inhibition effects. That is, cancer cells with similar mutation/copy number/expression profile have similar response profiles, and drugs with similar chemical properties/target proteins/ATC-codes have similar inhibition effects. The similarity between cells c and c′ under their response measurements was calculated by the Gaussian kernel based on their IC50 profile *μ* and *μ*′: 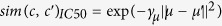, where *γ*_*μ*_ is pre-determined parameter. Meanwhile, the similarity between drugs d and d′ under their response measurements was calculated by the Gaussian kernel based on their IC50 profile *σ* and *σ*′ :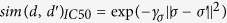, where *γ*_*σ*_ is pre-determined parameter.

**Figure 2:**
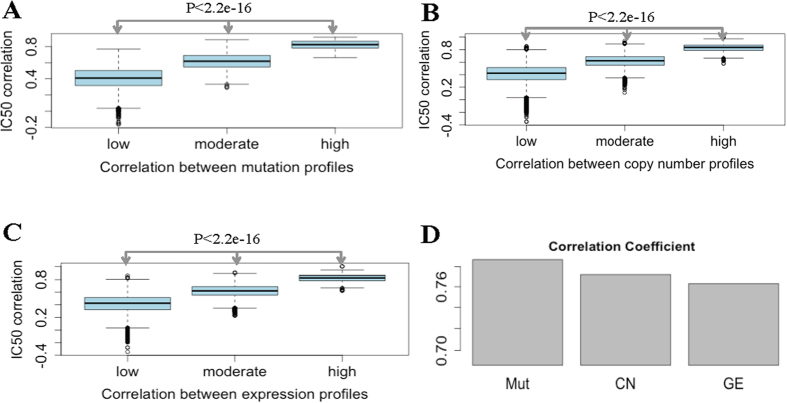
Correlating genomic features with cell response profiles to anti-cancer drugs. (**A–C**) The boxplots showing cells with similar genomic features responding to their IC50 correlations. X-axis indicates the correlations between cells under their genomic features, while y-axis indicates the correlations between cells under their IC50 profiles. (**D**) Barplots showing the PCCs between oncogene mutation, copy number alteration, and expression value and cell IC50 profiles. It shows that cell responses correlate mutation similarity more than other similarity measurements.

The IC50 correlations were significant higher for cells with more similar genomic features (Fig. 2A–C). The statistical differences among groups were calculated by the t-test and the p-value were less than 1e-16 for all three types of features. The over 0.7 Pearson Correlation Coefficients (PCCs) between IC50 and genomic features were shown in Fig. 2D as barplots. The p-values were less than 1e-3 for all three PCCs. These results mean that all mutation, copy number, and expression similarity correlate with IC50 correlations well. That is, cells with similar genomic features (mutation, copy number, and expression profiles) exhibit similar response profiles. Moreover, Fig. 2 showed that mutation correlated more with IC50 correlations, comparing with other two features. It not only displayed the highest correlation coefficient value, but also got highest IC50 correlations in cells with all low, moderate, and high similar genomic features. In another aspect, boxplots in Fig. S1 showed that drug sensitivity correlations were significant higher for drugs with more similar chemical and therapeutic properties. The over 0.3 PCCs were obtained and PCC between drug sensitivity and chemical property went beyond 0.5. That is, chemical property correlated more with drug response profile. All these results together suggest that all data sources about cancer cells and drugs are predictive. Furthermore, IC50 correlates more with cell mutation profile and drug chemical property, which indicates that cell mutation profile and drug chemical property may play important role during learning the association rules between cancer cells and drugs.

### Drug response prediction by PDRCC

**Figure 3:**
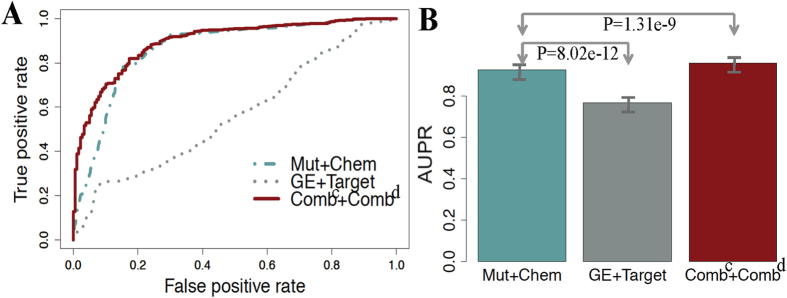
The ROC curves and AUPRs of PDRCC on various data source. (**A**) The ROC curves on the most predictive, the worst contributed data sources for cell and drug, and the combination of all data sources. (**B**) The AUPRs on the most predictive, the worst contributed data sources for cell and drug, and the combination of all data sources. It shows that the performance of cell-drug association identification can be significantly improved by combination of all data sources about cell and drug.

We firstly validated the performance of PDRCC on each single data source when utilizing the IC50 as the response measurement. The effect of mutation, copy number, and expression similarity on uncovering the observed cell-drug associations were shown by replacing the cell similarity matrix 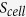 in kernel function (Method) with *S*_*Mut*_, *S*_*CN*_ and *S*_*GE*_, respectively. And the effect of chemical property, target protein, and ATC-code similarity on uncovering the observed cell-drug associations were shown by replacing the drug similarity matrix *S*_*drug*_ in kernel function (Method) with *S*_*Chem*_, *S*_*Target*_ and *S*_*ATC*_, respectively. The performance of each single data source on learning the association roles between cancer cells and drugs was evaluated and visualized by ROC curves^18^ and precision-recall curves^19^. The precision-recall curves were also introdcued here due to the unbalanced issue. That is, the number of resistant associations is always much larger than the number of sensitive associations. While the precision-recall curves is the better index to evaluate the prediction performance on imbalance data^19^. The AUCs on each data source effect were displayed in Table 1. It showed that, from cell side, “Mut” performed the best, and “CN” and “GE” achieved comparable prediction performance. From drug side, “Chem” achieved the best performance and “Target” performed the worst. Among all combination of data sources, the highest AUC of 0.798 was achieved by “Mut + Chem”, and the worst AUC of 0.572 was obtained by “GE + Target”. The precision-recall curves (Fig. S2) obtained by each single data source also indicate the best performance of “Mut + Chem”, and the worst performance of “GE + Target”. We drew ROC curves of “Mut + Chem” and “GE + Target” in Fig. 3A, it displayed that the ‘bad’ guy “GE + Target” could make the ROC curve beyond the diagonal (random classification), and the ‘good’ guy “Mut + Chem” made ROC close to 0-1 baseline. Moreover both two AUPRs were larger than 0.75 (Fig. 3B), suggesting the efficiency of all data sources on distinguish sensitive associations from the larger number of resistant associations. All these results together indicate that, each data source for cell and drug will do one’s bit in inferring the potential rules from the existing cell-drug associations. Therefore, combination of these data sources should produce a much more sophisticated picture of the associations among cells and drugs.

Table 1 suggested that, “Comb^c^” and “Comb^d^” performed better than using single data source, and most important thing was that the highest AUC of 0.89 and AUPR of 0.957 were obtained by integration of all data sources from both cell and drug sides. For example, “Mut + Chem” obtained an AUC of 0.792, while “Comb^c^ + Chem” and “Mut + Comb^d^” made the AUC 0.852 and 0.827, respectively; “GE + Target” obtained an AUC of 0.572, while “Comb^c^ + Target” and “GE + Comb^d^” made the AUC reach to 0.809 and 0.743, respectively, which has two percent improvement comparing with “GE + Target” did. In addition, the ROC curves (Fig. 3A) and precision-recall curves (Fig. S2) suggests that “Comb^c^ + Comb^d^” performed better than using mutation for cell and chemical property for drug, which was most predictive data sources among all single one for cell and drug. All these facts demonstrate that all data sources are useful in prediction. Combination of them significantly improves the accuracy of cell-drug association identification.

### Comparison with alternative integrative strategy

In this work, we integrated multiple properties of drugs, including chemical information, ATC-code annotation, and the drug target protein, and multiple genomic data sources of cancer cells, including somatic mutation, DNA copy number, and gene expression value. The maximum among them was applied to obtain good predictions. However, there are alternative strategies to address the same issue, such as the multiple kernel learning (MKL), which optimizes the weight to integrate kernels^20^,^21^,^22^. MKL is a unified framework and has elegant model to integrate different data sources. To achieve the prediction results through MKL, we implemented the MKL optimization procedure. That is, iteratively obtained the optimal weights to integrate kernels and the decision function. For saving the computational cost, we only validated MKL on either drug or cell side. That is, only using implemented MKL optimization procedure on either cell or drug side. Previous results indicated that somatic mutation and compound chemical properties provide more information in prediction, comparing with other data sources. Thus, for comparison, we implemented MKL on drug side and mutation similarity in cancer cell, and implemented MKL on cell side and chemical property similarity in drug. It turns out that MKL achieved the best AUC of 0.824 when implementing MKL in drug and using somatic mutation to represent cell similarity. This performance was comparable with “Mut + Comb^d^” did. All these results suggest that our simplified strategy is the better option for integrating data sources. In addition, MKL will add extra computational complexity. So in practice, it is better to choose the maximum strategy in our work to simplify the model and make it available to large-scale problems.

### Tissue specific conditions

**Figure 4:**
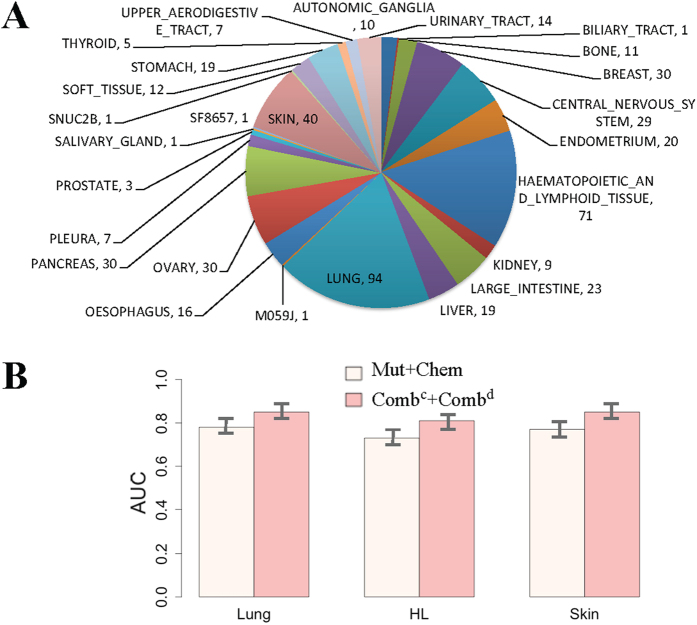
The performance of PDRCC under specific tissue conditions. (**A**) The distribution of tissue types in cancer cells in CCLE. The most majority tissues are Lung, haematopoietic, and lymphoid tissue (HL), and Skin. (**B**) The AUCs and AUPRs on Lung, haematopoietic and lymphoid tissue (HL), and Skin. It shows PDRCC could achieve the consistent performance on diverse tissues.

Proteins are dynamic in biological process. Their function may vary in different tissues and conditions. This fact would influence the drug responses in diverse tissue types. Therefore, the diverse tissue conditions should be considered when validation the performance of PDRCC. The distribution of tissue types in the 504 cancer cells with responses available was shown in the Fig. 4A. The most major types were Lung, haematopoietic and lymphoid tissue (HL), and Skin. They were taken 18% (90), 14% (71), and 8% (40) of all 504 cancer cells, respectively. We validated the effect of PDRCC on discovery the association between drugs and the cells in each of above three tissues. The AUC and AUPR obtained by “Comb^c^ + Comb^d^” were shown as barplot in Fig. 4B. The AUCs and AUPRs obtained in all three tissues were above 0.75 and 0.80, respectively. In addition, for all three tissues, PDRCC always achieved higher AURPs than AUCs, suggesting that PDRCC is suitable to distinguish the sensitive associations from those larger numbers of resistant associations under specific tissue condition. These results indicate the efficiency of our PDRCC on different tissues. Moreover, both AUCs and AUPRs were not varied too much in three tissue types. For example, the AUCs were 0.78, 0.75, and 0.77 on Lung, HL, and skin, respectively; the AUPRs were 0.83, 0.81, and 0.82 on Lung, HL, and skin, respectively. These results together suggest that our PDRCC can not only discovery the association between drugs and the cells with different tissue types, but also achieve the consistent accuracy on diverse tissue types.

### The efficiency of PDRCC in uncovering diverse measurements of drug response

**Figure 5:**
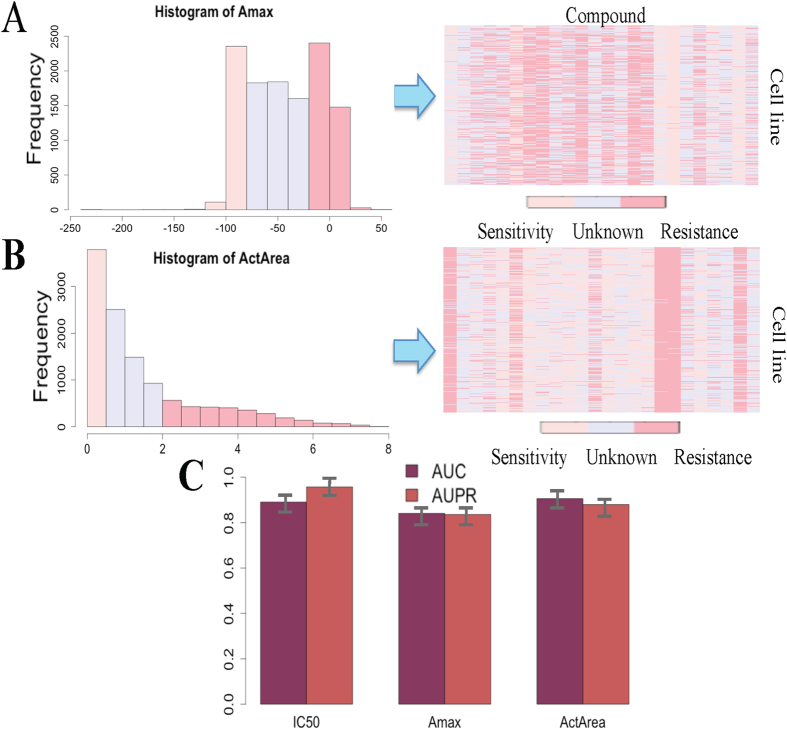
The performance of PDRCC on diverse measurements of drug response. (**A**) Assigning Amax into three classes: sensitive, resistant and unknown. (**B**) Assigning ActArea into three classes: sensitive, resistant and unknown. (**C**) The AUCs and AUPRs obtained based on the diverse measurements of drug response. PDRCC produces the consistent performance when measurements of drug response changing.

Previous analysis indicated that PDRCC performed well in predicting associations between cancer cells and drugs based on IC50 measurement. To test whether it can produce the consistent performance on another measurements of drug response, such as the maximal activity value (Amax) and the area between the drug-response curve and a fixed reference (ActArea), the PDRCC was performed on above two measurements, respectively. Specifically, the value of Amax and ActArea were firstly discrete into three categories: sensitive, resistant, and unknown (Fig. 5A,B). Then the combination kernel of cancer cells and drugs were applied to integrate cell genomic features and drug chemical and therapeutic properties. Finally, the 10-fold cross-validation was done to validate the performance of PDRCC.

The effect of PDRCC on uncovering the observed cell-drug associations based on Amax and ActArea measurements were shown by AUC and AUPR in Fig. 5C. Although the AUCs obtained based on both Amax and ActArea were lower than the IC50 did, they were larger than 0.8. It suggests the efficiency of PDRCC in other types of response measurements. We have to note that the prediction problem when either using Amax or ActArea as the measurement of drug response is imbalance due to the inequality number of resistant and sensitive associations. For IC50, AUPR was larger than AUC. While, for both Amax and ActArea, “Comb^c^ + Comb^d^” achieved comparable AUPR and AUC, and both AUPRs were larger than 0.75. This result means that when using both Amax and ActArea to measure the response value, PDRCC is effective in distinguishing the sensitive associations from the different number of resistant associations. These results together confirm the efficiency of PDRCC by using different gold-standard datasets is indicated by above results.

### Novel prediction

By cross-validation, PDRCC displayed its promising performance in predicting observed cell-drug associations, especially using IC50 to measure the drug response. To test whether it could produce biologically useful predictions, we focus on the unknown cell-drug pairs, which were obtained by categorizing method. We trained “Comb^c^ + Comb^d^” on the sensitive and resistant associations, and tested it on 2,774 unknown cell-drug pairs. Since we may be more interested in the discovering the novel sensitive associations between cancer cells and anticancer therapies, thus our expectation is that “Comb^c^ + Comb^d^” can discover novel sensitive associations between cancer cells and drugs.

The top five sensitive associations were listed in Table 2. For each novel prediction, we searched the database and literature evidences from CCLE and PubMed to support the efficiency of PDRCC in uncovering the novel sensitive associations between cancer cells and known anti-cancer drugs. Taken the top one prediction as an example, the cell subtype of cancer cell ‘A549_LUNG’ is Non Small Cell Lung Cancer (NSCLC), and the literatures^23^,^24^,^25^,^26^,^27^ indicated that the anti-cancer drug ‘Topotecan’ was ready to be the novel therapeutic strategy in the treatment of NSCLC and Small Cell Lung Cancer (SCLC). These evidences support the sensitive association between ‘A549_LUNG’ and ‘Topotecan’. The similar story for remaining four novel predictions can be addressed from Table 2. In conclusion, database and literature search support these novel predictions. That is, PDRCC can uncover potential sensitive drugs to caner cells, which provide candidates for further experiments.

## Discussion

Systematical approach to identify the novel associations between cancer cells and anti-cancer therapies may guide the early-phase clinical trials of multiple novel compounds under development. Here, we proposed a novel systematical approach to predict responses of multiple drugs in hundreds of cancer cells simultaneously in one model by inferring the associations between cancer cells and drugs. This strategy make our prediction model can be applied not only to predict the response of a newly measured cell to already tested drugs, but also to predict the inhibition effect of an existing drug in cancer cells with known genomic information. It would greatly save the cost in drug-cell screening. The machine learning framework was constructed to implement the prediction task and the kernel method was applied to integrate pharmacogenomics data. Our main contributions here are both in proposing the machine learning framework and integrating heterogeneous data from both cell and drug sides through kernel function to construct the predictive model. The validity of this approach, called as PDRCC, was supported by its effective in uncovering the cell-drug associations with database and literature evidences, and it set the stage for clinical testing of novel therapeutic strategies, such as the sensitive association between cancer cell ‘A549_LUNG’ and compound ‘Topotecan’. Database and literature searching indicate that our novel inhibitors provide the promising opportunities to cure their predicted sensitive cancer cells. In conclusion, PDRCC will hopefully enhance the discovery and validation of additional predictive cancer therapeutics. Here we only attempted to improve the accuracy of drug response prediction. However, the biomarkers, that determine the sensitive and resistant association between cancer cells and anti-cancer therapies, are urgently needed in clinical applications. Thus the future work will extent this work to include the biomarker determination, to make the prediction algorithm not only produce the promising associations between cancer cells and therapies, but also uncover the novel biomarkers of sensitivity and resistance to cancer therapeutics.

In this work, instead of learning the exact response value, which usually did in previous work^6^,^8^,^9^,^10^, we studied drug response by detecting the binary relationships (sensitive or resistant) between cells and drugs. It is not only because of the inaccuracy of experimentally measured response value, but also because that people may have more interests on whether the cancer cell is sensitive or resistant to a given therapy than what the exact the response value is. While we also noted that, by casting continuous IC50s into discrete ones, our prediction task became imbalance, due to unbalanced number of sensitive associations and resistant associations. SVM model took care of this issue by assigning different weights to sensitive associations and resistant associations, respectively, and the good performance was achieved. To show the importance of dealing with the imbalance issue during learning, we compared our method with other machine learning algorithms, such as logistic regression and random forests, which are more interpretable for the question that where the prediction coming from. We firstly run logistic regression (non specific strategy for unbalanced data) on our integrated datasets, and applied 10-fold cross-validation to validate the performance. Turns out that, the logistic regression achieved about 0.7 AUC and 0.6 AUPR, which were worse than “Comb^c^ + Comb^d^” obtained. Furthermore, it obtained less than 0.5 true positive rate and about 0.7 true negative rate, suggesting that the worse performance may come from the ignoring the unbalanced issue. Then, we validated the performance of random forests (addressing imbalance issue through incorporating diverse class weights) on our integrated datasets through 10-fold cross-validation. As a matter of fact, random forests achieved an AUC of 0.919 and AUPR of 0.898, respectively. While, “Comb^c^ + Comb^d^” obtained an AUC of 0.89 and 0.957, respectively. Although, AUC is higher, AUPR (the better index to evaluation the performance of classifier on imbalance problem) is much lower. All these results suggest the importance for dealing with unbalanced issue during learning, and our PDRCC is suitable to distinguish sensitive responses from different number of resistant responses of drugs.

Previous work applied various data sources to describe drug’s biological function from different levels and aspects. Here, the chemical properties, therapeutic annotations and effects were utilized to measure the similarity among drugs. We have to note that there are another data sources to describe the function of drugs, such as drug side-effects, which hint the unwanted effects of drug at phenotype level. Furthermore, previous work suggested the validity of drug side-effects in predicting drug mode of actions, including targeting proteins^28^, cured diseases^29^,^30^,^31^ *et al.* Therefore, drugs with similar side-effects may indicate the similar profile of responses in cancer cells. However, there are only few of 24 compounds in CCLE with their side-effects available. For example, only six compounds got their side-effects in SIDER database (http://sideeffects.embl.de/). Previous work indicated the strong associations between drug side-effects and target proteins^28^, thus we already included side-effects information in some sense by introducing target proteins to represent the similarity among drugs.

Here, we utilized sequence information to characterize the similarity among proteins, and the drug similarity under protein target measurement was then defined by the maximum sequence similarities among their target proteins. The experimental results showed that sequence information was predictive in drug response prediction. One concern is that protein sequence similarity is too strict to measure the similarity among proteins. Because two proteins may be similar to each other due to another reason, such as they are co-expressed and have some functional linkage^32^,^33^. In future, we will extend our work to include other data sources for protein in drug response prediction. For example, we could define the protein similarity through GO annotation and expression value *et al.* Another possible improvement might be to use the defined interacting domain in protein sequence and to make the sequence similarity score more accurate.

Besides cell genomic features and drug chemical and therapeutic features, there are other types of features were applied to detect the drug responses in cancer cells. For example, Majumder *et al.* integrated the tumor ecosystems with a novel machine learning algorithm to predict the therapeutic efficacy of targeted and cytotoxic drugs in patients with head and neck squamous cell carcinoma (HNSCC) and colorectal cancer (CRC)^34^; Frieboes *et al.* attempted to implement a novel quantitative approach to study the drug effects on the growth and regression of tumor mass based on cell phenotype^35^. All these informative data sources could be easily incorporated into our model. In future, we will try to incorporate much more data sources from both cell and drug sides to further improve the accuracy of our prediction model.

### Methods and Materials

Given two cancer cell and drug pairs, we considered to construct a kernel function, which potentially correlated with them. Since the kernel function represents the similarities among the training samples in some sense^36^, we focused on the similarity scores among samples rather than the sample profile itself for each data source.

### Cancer cell similarity

The oncogene mutation, DNA copy number, and mRNA expression were applied to calculate the similarity among cells.

### Oncogene mutation

CCLE provided 25 oncogene mutations across 486 cancer cells. The mutation MAF file was used for somatic mutation data analysis. A gene-by-sample matrix of binary values (1-mutated, 0-wildtype) was generated for similarity calculation. The matrix *S*_*Mut*_ was applied to represent the cell similarity matrix based on their oncogene mutation measurement. Each row (or column) was the mutation based similarity profile for a single cell. The element of *S*_*Mut*_ was defined as the weighted cosine correlation coefficient: 
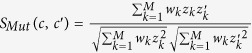
, where *z* and *z*′ are binary vectors for cell *c* and *c*′ representing the mutation or wide-type of the corresponding oncogene. *w*_*k*_ is the weight for the k-th oncogene, defined as 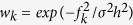, where *f*_*k*_ is the mutation rates of the k-th oncogene in the data and *M* is the total number of oncogene (equals to 25 here), *σ* is the SD of 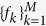, and h is a parameter (set to 10 in this study).

### DNA copy number

There were 23,316 gene copy numbers across a total of 1043 cancer cells based on CCLE ‘CCLE_copynumber_byGene_2013-12-03’ TXT file. Given two cells c and c′, the copy number based similarity between them was calculated by Gaussian kernel function. A matrix 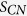 was then constructed to represent the copy number similarity for cancer cells. Each row (or column) of this matrix was the copy number based similarity profile for a single cell.

### mRNA expression

There were 54,675 gene expression values across a total of 127 cells based on CCLE ‘CCLE_Expression_2012-09-29’ CSV file. Given two cells c and c′, the gene expression based similarity between them was calculated by the absolute value of Pearson Correlation Coefficient between their gene expression values across the CCLE cells. A matrix *S*_*GE*_ was then constructed to represent gene expression similarity for cell lines. Each row (or column) of this matrix was the expression based similarity profile for a single cell.

### Drug similarity

The compound chemical properties, drug ATC-codes, and drug-targets were used to represent the similarity among drugs, respectively.

### Compound chemical properties

The compound chemical property for each drug came from a collection of molecular descriptors was calculated by QuaSAR-Descriptor in the Molecular Operating Environment (MOE v. 2011.10, Chemical Computing Group Inc., Montreal, Canada). The MOE descriptor generated a total of 308 features for 24 compounds, which included 2D descriptors, Internal 3D descriptors, and External 3D descriptors. Then the chemical similarity between two drugs d and d′ was computed by the Gaussian kernel function on their molecular descriptors. A matrix *S*_*Chem*_ was then constructed to represent chemical similarity for drugs. Each row (or column) of this matrix was the chemical property similarity profile for a single drug.

### Drug-targets

The target proteins for 24 compounds were provided by CCLE. Given two drugs d and d′, the target-based similarity between them was calculated as follows: 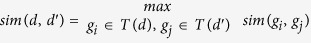, where *T*(*d*) and *T*(*d*′) are the sets of target proteins for *d* and *d*′, respectively. The sequence data was applied to measure protein similarity due to the rapidly developed sequencing techniques. The sequence similarities among proteins were defined by a normalized version of Smith-Waterman scores^37^. They were calculated by “swalign” function in Matlab Bioinformatics toolbox. A matrix *S*_*Target*_ was then constructed to represent target protein similarity for drugs. Each row (or column) of this matrix was the target protein similarity profile for a single drug.

### ATC-codes

ATC-codes of drugs were extracted from WHOCC. Considering the hierarchical structure of ATC-codes, a probabilistic model^38^ was introduced to calculate the similarity. Specifically, the similarity between two ATC-codes (*t*_*i*_ and *t*_*j*_) was calculated as follows: 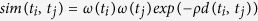, where *d*(*t*_*i*_, *t*_*j*_) is the shortest distance between ATC-codes *t*_*i*_ and *t*_*j*_ in the hierarchical structure of the ATC classification system, *ω*(*t*_*i*_) and *ω*(*t*_*j*_) represent the weights of the corresponding ATC-codes, and were defined as the inverse of ATC-code frequencies, which means that more emphasis was put on specific codes rather than the general ones^39^. *ρ* is a predefined parameter (set to be 0.25 in this study). The drug ATC-codes similarity was calculated by the equation of 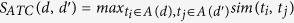, where *A*(*d*) and *A*(*d*′) are the sets of ATC-codes for 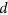
and *d*′, respectively. *S*_*ATC*_ was used to denote the resulting drug ATC similarity matrix. Each row (or column) of this matrix was the ATC-code annotation similarity profile for a drug.

### The kernel function for data fusion

With the representation of drugs and PPIs by their similarity profiles, the kernel function with cell-drug pairs was calculated as Kronecker product kernel^40^,^41^: 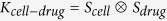, where *S*_*cell*_ can be any one of *S*_*Mut*_, *S*_*CN*_ and *S*_*GE*_ or their combination and *S*_*drug*_ can be any one of *S*_*Chem*_, *S*_*Target*_ and *S*_*ATC*_ or their combination.

In this paper, “Mut” denoted the case when *S*_*cell*_ = *S*_*Mut*_, “CN” denoted the case when *S*_*cell*_ = *S*_*CN*_, “GE” denoted the case when *S*_*cell*_ = *S*_*GE*_, and “Comb^c^” denoted the case when *S*_*cell*_=max(*S*_*Mut*_, *S*_*CN*_, *S*_*GE*_), which means cell similar in one or more than one metrics will sensitive/resistant to similar drugs. “Chem” denoted the case when *S*_*drug*_ = *S*_*Chem*_, “Target” denoted the case when *S*_*drug*_ = *S*_*Target*_, “ATC” denoted the case when *S*_*drug*_ = *S*_*ATC*_, and “Comb^d^” denoted the case when *S*_*drug*_ = max(*S*_*Chem*_, *S*_*Target*_, *S*_*ATC*_), which means drug similar in one or more than one metrics will have similar therapeutic effects. “Comb^c^ + Comb^d^” denoted the case when *S*_*cell*_ = max(*S*_*Mut*_, *S*_*CN*_, *S*_*GE*_) and *S*_*drug*_ = max(*S*_*Chem*_, *S*_*Target*_, *S*_*ATC*_). Taken together, the rationale behind our kernel function construction scheme for cell-drug pairs is that two cell-drug pairs are similar only when the corresponding cell and drug are simultaneously similar supported by different lines of evidences.

### Prediction of drug response by using the defined kernel function and a ‘categorical’ classifier

With the above kernel function construction scheme, the drug response prediction task was ready to feed to SVM. Here, we’d like to apply a ‘categorical’ classifier to implement prediction task. That is, instead of estimating the continuous response value, we assigned response value into the classes of sensitive, resistant and unknown, and predicted whether cancer cell is sensitive or resistant to anti-cancer therapy.

To this end, we first drew a distribution of response values (IC50: the half maximal inhibitory concentration of a substance with respect to cell viability), and then categorized them into three classes: sensitivity, resistance, and unknown. The distribution of IC50 values across all 504 cell lines were drawn in the left picture of Fig. S3. Obviously, there were three types of bars in the histogram. They were the bars for value range from 0 to 0.5, 0.5 to 7.5, and 7.5 to 8, respectively. The two dramatic bars were used to determine the classes of sensitivity/resistance, that is, sensitivity was for those IC50s changing from 0 to 0.5, and resistance was for those IC50s form 7.5 to 8. Under this setting, IC50 profiles across cell lines were then become a relationship matrix with three values: 1, 0, −1, which represented sensitivity, unknown, and resistance relationship, respectively (right panel of Fig. S3). After above categorizing, drug response prediction problem was ready to be formalized as a binary classification problem with a pair of cell and drug as prediction input, sensitive or resistant relationship between them as the output, which was feeding to SVM-based algorithms^15^,^16^,^42^.

### Benchmark datasets and SVM implementation

The dataset used to validate our method came from CCLE, which contains 8-point dose-response curves for 24 compounds across 504 human cancer cell lines. The sensitive and resistant associations between cancer cells and drugs were utilized as gold-standard positive and negative dataset, respectively. Oncogene mutation, DNA copy number, and mRNA expression were applied to represent cell lines, which came from CCLE ‘CCLE_Oncomap3_2012-04-09’ MAF file, ‘CCLE_copynumber_byGene_2013-12-03’ TXT file, and ‘CCLE_Expression_2012-09-29’ CSV file, respectively. Chemical properties, drug-targets, and ATC-code annotations were utilized to measure the similarity among drugs. Chemical property came from a collection of molecular descriptors calculated by QuaSAR-Descriptor in the Molecular Operating Environment (MOE v. 2013.10, Chemical Computing Group Inc., Montreal, Canada). Target protein amino acid sequences were extracted from UniProt (http://www.uniprot.org/). ATC-codes of drugs were extracted from World Health Organization Collaborating Centre (WHOCC) (http://www.whocc.no/atc_ddd_methodology/who_collaborating_centre/).

We trained the SVM-based predictor by using LibSVM^43^. In our implementation, the penalty parameter C was optimized by grid search approach with 3-fold cross-validation, and the optimal value of C was 10. To evaluate the performance of PDRCC, 10-fold cross-validation was introduced here. The performance of PDRCC was shown by receiver operating characteristic (ROC) curve^18^, which shows the trade-off between the true positive (correctly predicted interactions) rate (TPR) with respect to the false positive (wrongly predicted interactions) rate (FPR). We noted that our prediction task was imbalance, because that the number of resistant associations was usually much larger than the number of sensitive association about three times. For example, there were 2,564 sensitive associations when using IC50 as the measurement of drug response. While, the number of resistant associations was 6,750, which were about three times of the number of sensitive ones. Thus, we introduced the precision-recall curve^19^, which is the better index to evaluation the performance of classifier on imbalance problem, to further evaluate the performance of our PDRCC. Furthermore, the evaluation criteria, area under ROC (AUC), and area under precision-recall curve (AUPR) were also used to assess the performance of the proposed predictive methods.

## Additional Information

**How to cite this article**: Wang, Y. *et al.* Inference of drug responses in cancer cells from cancer genomic features and compound chemical and therapeutic properties. *Sci. Rep.* **6**, 32679; doi: 10.1038/srep32679 (2016).

## Supplementary Material

Supplementary Information

## Figures and Tables

**Table 1:** The AUC obtained by PDRCC by considering all of data sources separately, two together, and all together.

AUC	Chem	ATC	Target	Comb^d^
Mut	0.798 ± 0.01	0.776 ± 0.008	0.752 ± 0.007	**0.827** ± **0.006**
CN	0.618 ± 0.008	0.613 ± 0.005	0.582 ± 0.008	**0.713** ± **0.005**
GE	0.62 ± 0.007	0.607 ± 0.007	0.572 ± 0.01	**0.743** ± **0.006**
Comb^c^	**0.852** ± **0.007**	**0.846** ± **0.006**	**0.809** ± **0.005**	**0.89** ± **0.005**

The best predictions obtained are highlighted in bold.

**Table 2:** The top five novel sensitive associations obtained by PDRCC on “Comb^c^ + Comb^d^” kernel and the value of IC50.

Rank	Cell	Drug	Cell Type	Drug Usage
1	A549_LUNG	Topotecan	NSCLC	NSCLC^23^,^24^, SCLC^25^,^26^,^27^
2	MFE319_ENDOMETRIUM	17-AAG	Endometrioid adenocarcinoma	Endometrial Carcinoma^44^,^45^
3	K029AX_SKIN	Irinotecan	Malignant melanoma	Melanoma^46^,^47^
4	MIAPACA2_PANCREAS	Irinotecan	Ductal carcinoma	Pancreas^48^,^49^
5	MDAMB453_BREAST	Nilotinib	—	Tamoxifen-resistant breast cancer^50^

The abbreviation: Non Small Cell Lung Cancer (NSCLC), Small Cell Lung Cancer (SCLC).
