# Supplementary material for: Inferences of drug responses in cancer cells from cancer genomic features and compound chemical and therapeutic properties
Source: Sci Rep. 2016 Sep 20;6:32679. doi: 10.1038/srep32679 (PMC5028846; doi:10.1038/srep32679)
Supplement: Supplementary Information [file srep32679-s1.pdf]

Supplementary Materials

Inference of drug responses in cancer cells from cancer genomic features and compound chemical and therapeutic properties

Yongcui Wang[1@*], Jianwen Fang[2@], Shilong Chen[1*]

1, Key Laboratory of Adaptation and Evolution of Plateau Biota, Northwest Institute of Plateau Biology, Chinese Academy of Sciences, Xining, China, 810001.

2, Biometric Research Branch, Division of Cancer Treatment and Diagnosis, National Cancer Institute, Rockville, MD 20850, USA.

[@] These authors contributed equally to this work.

* Correspondence: ycwang@nwipb.cas.cn (Y.C.W), slchen@nwipb.cas.cn (S.L.C.).

Legend

Fig S1

Correlating compound properties with drug response profiles. The boxplots show drugs with similar chemical features correlation well with IC50 profiles. X-axis indicates the correlations between drugs under their chemical and therapeutic properties, while y-axis indicates the correlations between drugs under their IC50 profiles. Barplots show the PCCs relating chemical property, target proteins, and ATC-codes with response profiles under IC50 measurement. It shows that drug responses correlate chemical property similarity more than other similarity measurements.

Fig S2

Precision-recall curves on each single data sources and their combinations. The precision-recall curves on each single data sources and their combinations are displayed with difference colors, it shows oncogene mutation and chemical properties are most predictive data sources, and expression and target protein might play trivial roles in prediction. The improved performance can be obtained by integrating all data sources of cells and drugs.

Fig S3

Assigning response value into three classes: sensitive, resistant and unknown. The left picture shows the distribution of IC50s across all 504 cell lines. Three kinds of bars are shown with different colors, and it determines three status of drug response: sensitivity, resistance and unknown relationship. The right picture is the heat map of relationship matrix. The horizontal axis represents drugs, and the vertical axis represents cell lines.

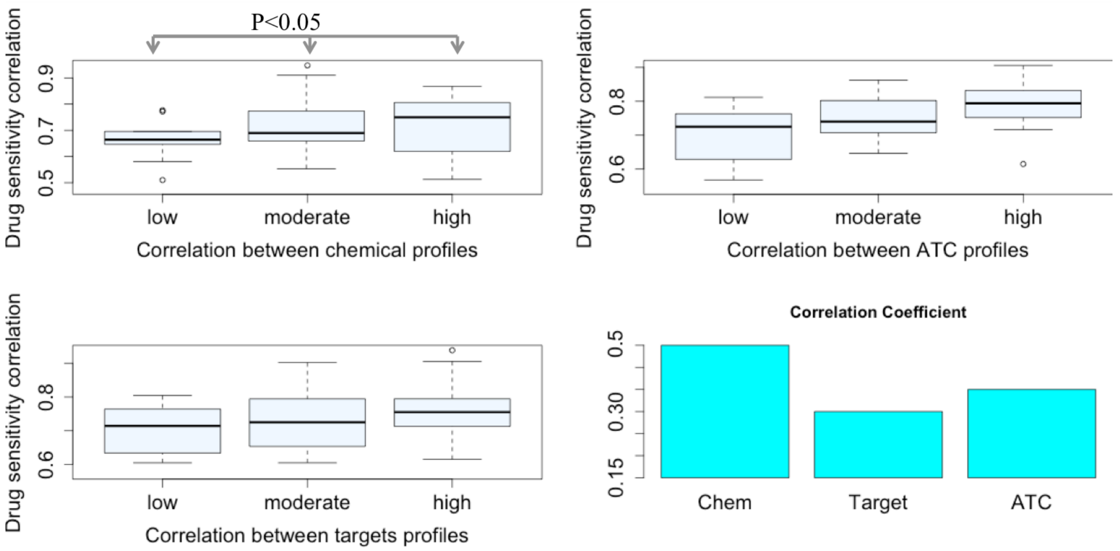
P<0.05
Drug sensitivity correlation
0.5
0.7
0.9
low
moderate
high
Correlation between chemical profiles
Drug sensitivity correlation
0.6
0.8
low
moderate
high
Correlation between ATC profiles
Drug sensitivity correlation
0.6
0.8
low
moderate
high
Correlation between targets profiles
Correlation Coefficient
0.15
0.30
0.5
Chem
Target
ATC

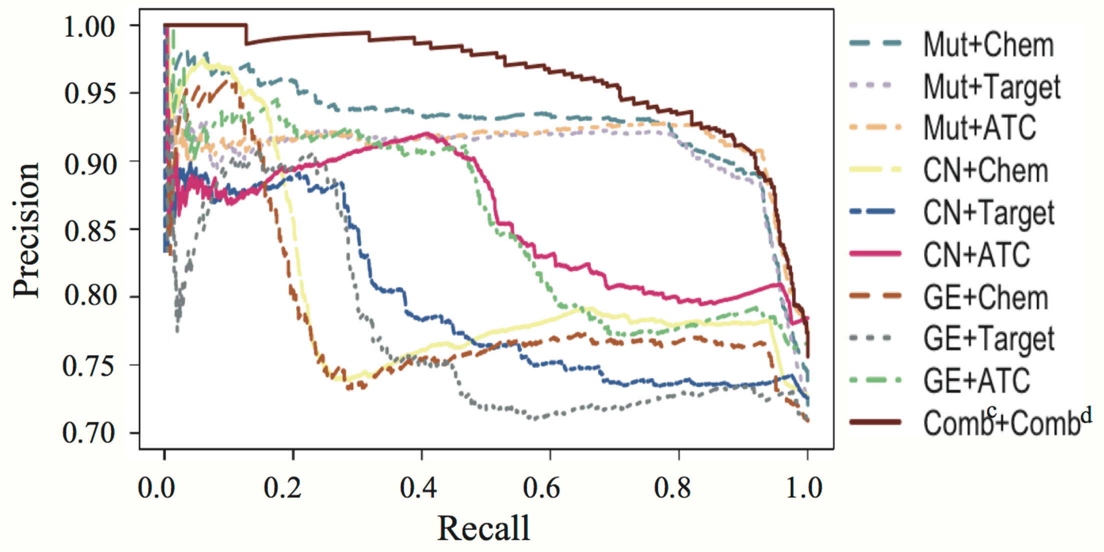
Precision
1.00
0.95
0.90
0.85
0.80
0.75
0.70
0.0
0.2
0.4
0.6
0.8
1.0
Recall
Mut+Chem
Mut+Target
Mut+ATC
CN+Chem
CN+Target
CN+ATC
GE+Chem
GE+Target
GE+ATC
Comb^c+Comb^d

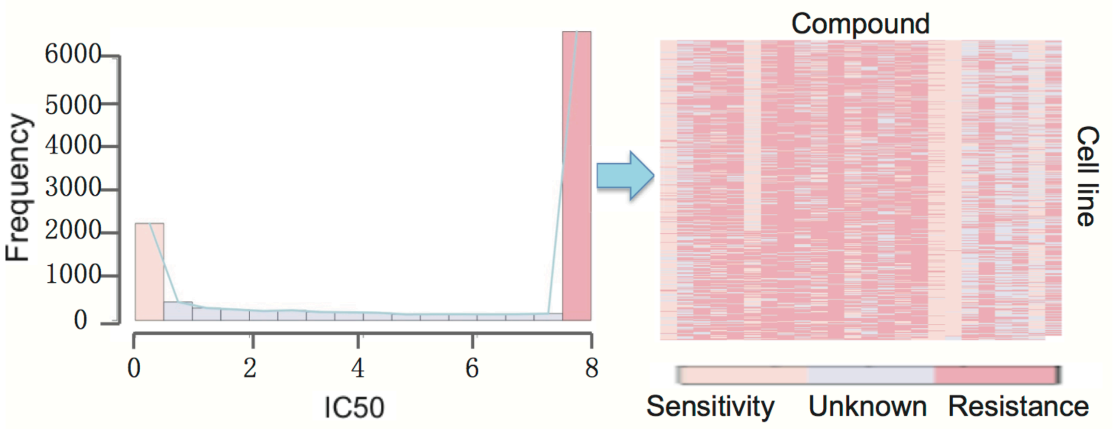
Frequency
6000
5000
4000
3000
2000
1000
0
0
2
4
6
8
IC50
Compound
Cell line
Sensitivity
Unknown
Resistance
